# Programmable Real-time Clinical Photoacoustic and Ultrasound Imaging System

**DOI:** 10.1038/srep35137

**Published:** 2016-10-12

**Authors:** Jeesu Kim, Sara Park, Yuhan Jung, Sunyeob Chang, Jinyong Park, Yumiao Zhang, Jonathan F. Lovell, Chulhong Kim

**Affiliations:** 1 Departments of Creative IT Engineering and Electrical Engineering, Pohang University of Science and Technology (POSTECH), 77 Cheongam-Ro, Nam-Gu, Pohang, Gyeongbuk, 37673, Republic of Korea; 2 Alpinion Medical Systems, 72 Digital-Ro 26-Gil, Guro-Gu, Seoul, 08393, Republic of Korea; 3 Department of Biomedical Engineering, University at Buffalo, The State University of New York, Buffalo, NY 14260, United States of America

## Abstract

Photoacoustic imaging has attracted interest for its capacity to capture functional spectral information with high spatial and temporal resolution in biological tissues. Several photoacoustic imaging systems have been commercialized recently, but they are variously limited by non-clinically relevant designs, immobility, single anatomical utility (e.g., breast only), or non-programmable interfaces. Here, we present a real-time clinical photoacoustic and ultrasound imaging system which consists of an FDA-approved clinical ultrasound system integrated with a portable laser. The system is completely programmable, has an intuitive user interface, and can be adapted for different applications by switching handheld imaging probes with various transducer types. The customizable photoacoustic and ultrasound imaging system is intended to meet the diverse needs of medical researchers performing both clinical and preclinical photoacoustic studies.

Photoacoustic imaging (PAI) is a hybrid imaging technique that integrates optical and ultrasound imaging (USI). PAI is based on the photoacoustic (PA) effect, which generally involves the following sequence of five events: (1) A pulsed laser illuminates a target object. (2) The object absorbs energy and expands thermoelastically. (3) The expansion resolves to its original size, generating PA waves which propagate in all directions. (4) The PA waves are detected by an acoustic wave detector. (5) PA images are reconstructed from the detected PA waves through image processing^1^,^2^. Based on intrinsic biological optical contrast (e.g., oxy-hemoglobin, deoxy-hemoglobin, and melanin), PAI provides functional and structural information about normal and abnormal tissues and biological phenomena such as angiogenesis, hemoglobin oxygen saturation, total hemoglobin concentration, and blood flow^3^,^4^,^5^. Furthermore, exogenous agents can enhance the contrast of the PA images and provide molecular information^6^,^7^,^8^,^9^,^10^. PAI inherits advantages from both USI and optical imaging, such as safety, economy, ease of implementation, and high temporal and spatial resolution. In addition, PAI can overcome drawbacks of both modalities: PAI offers dramatically deep penetration compared to pure optical imaging and rich optical contrasts compared to USI.

Because the signal reception mechanism of PAI is identical to that of USI, they can share an ultrasound (US) receiving system and transducer, so it is straightforward to adapt these two imaging modalities into an integrated imaging system^11^,^12^,^13^. In the integrated system, PAI and USI can respectively provide complementary information about functional optical absorption spectra and acoustical morphological structure in biological tissues. This complementary information from the integrated PA and US imaging (PAUSI) system can enhance diagnostic accuracy and benefit patients by reducing the time, discomfort, and cost of diagnosis and treatment.

These compelling features of PAUSI have driven the recent development of several commercial systems, whose specifications are compared in Table S1. First, a series of programmable USI systems (Verasonics, USA)^14^,^15^,^16^, which are amongst the most advanced research platforms, have been used in various PA applications by modifying the specific operation of the system using Matlab-based software. However, the Verasonics systems are generally US research machines rather than clinical systems for PAI, thus additional approval from the United States Food and Drug Administration (FDA) would be required to utilize the systems in clinics. The VevoLAZR (FujiFilm VisualSonics, Canada)^17^,^18^,^19^, Nexus 128 (Endra Life Science, USA)^20^,^21^,^22^, and MSOT Acuity (iThera Medical, Germany)^21^,^22^,^23^ are PAUSI systems which provide real-time volumetric PAUS images, as well as cross sectional images. They can also provide functional information, such as hemoglobin oxygen concentration and contrast enhancement from exogenous agents. However, the VevoLAZR equips high-frequency US transducers for small animal research, and thus the penetration depth is rather shallow for clinical applications. Further, the VevoLAZR system does not provide a programmable sequence. The Nexus 128 is mainly intended for small animal research, not for clinical applications. Likewise, typical MSOT systems are for preclinical applications. Among them, the MSOT Acuity is designed for exploratory clinical uses, but is not suitable for use as a general US imaging platforms. The LOUISA 3D (TomoWave, USA)^24^,^25^,^26^, PA mammography device (Canon, Japan)^27^,^28^,^29^,^30^, Twente Photoacoustic Mammoscope (University of Twente, Nederland)^31^,^32^, and Imagio (Seno Medical Instruments, USA)^33^ are specifically designed for breast cancer research, and to visualize vasculature, skin, or tumors using an arc-shaped, hemispherical, flat, and linear array transducer, respectively. However, these four systems are limited by either immobility or their breast-specific design. Particularly, the Imagio provides a handheld operation by using a linear array transducer and two fixed optical wavelengths. However, its application may be limited due to the lack of programmability and fixed excitation laser wavelengths. The Prodigy (S-Sharp, Taiwan)^34^, Sonix Touch Q+ (bk ultrasound, USA)^35^,^36^, and Z One PRO (Zonare Medical Systems, USA)^37^,^38^ are USI systems that provide a user interface for modifying transmitting/receiving sequences. The Prodigy provides a research platform for various transducers, but is intended for research use and is not a clinical device. The Sonix Touch Q+ and Z One PRO have potential for clinical PAUSI due to their programmable platforms and clinically oriented designs. However, a readily accessible interface for PAI is not presently available for these systems.

Here, we report a real-time clinical PAUSI system which consists of an FDA approved programmable clinical ultrasound machine and a portable laser system. The system is the first clinical PAUSI system built on a general purpose USI system. Real-time PAUS images are visualized in the US machine, which performs all data acquisition, image processing, and display tasks. The system has an intuitive user interface and a programmable research platform, so users can easily design and apply their own imaging algorithms. A programmable and clinically-ready research platform with switchable handheld probes makes this system directly suitable for studying various clinical concerns, such as thyroidal, hepatic, cardiac, intestinal, and ovarian diseases.

## Results

### Real-time clinical photoacoustic and ultrasound imaging system

**Figure 1:**
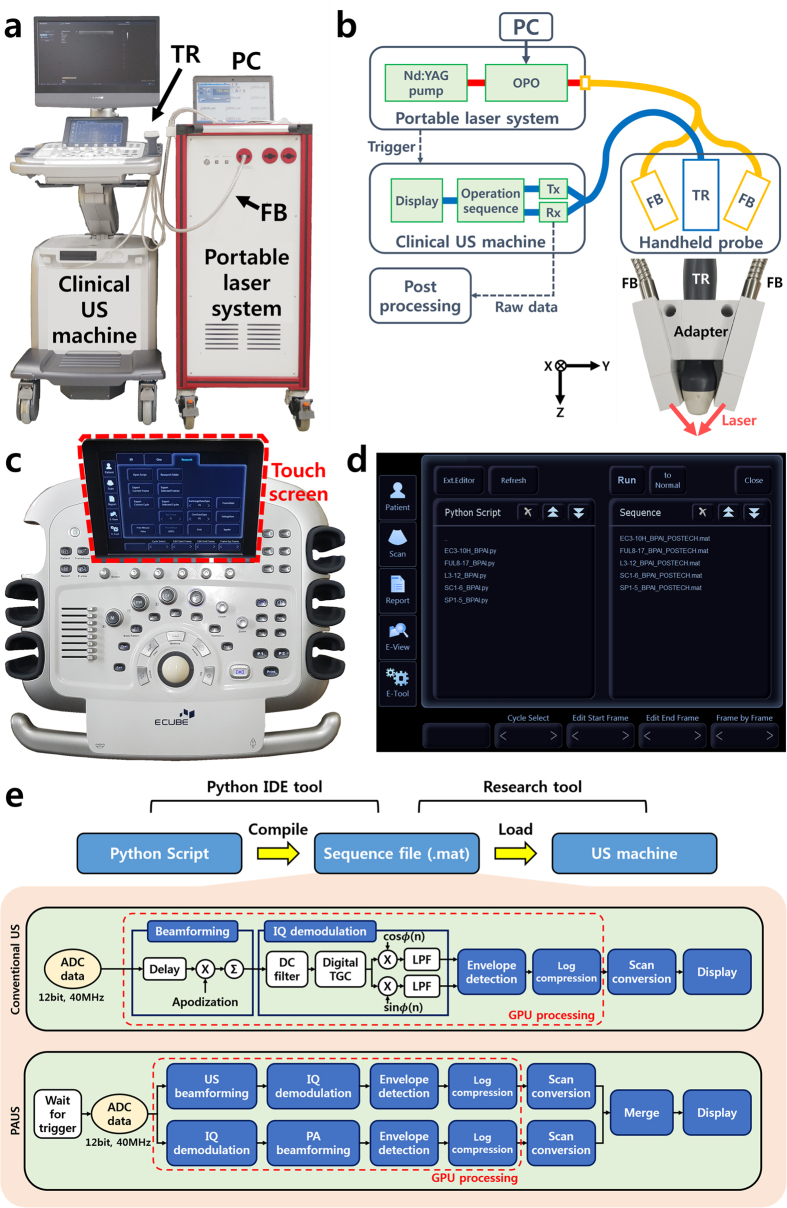
Photograph (**a**) and schematic (**b**) of the developed real-time clinical PAUS imaging system. (**c**) Photograph of the control panel. (**d**) Program selection user interface on touch screen pad. (**e**) Operation sequences of conventional ultrasound and PAUS imaging mode in the programmable US imaging system. PA, photoacoustic; US, ultrasound; TR, ultrasound transducer; FB, fiber bundle; PC, personal computer; OPO, optical parametric oscillator; BF, beamforming; Tx, transmitter; Rx, receiver; IDE, integrated development environment; ADC, analog to digital converter; DC, direct current; TGC, time gain compensation; and GPU, graphics processing unit.

Figure 1a is a photograph of the developed real-time clinical PAUSI system. The system consists of an FDA approved commercial programmable US machine (EC-12R, Alpinion Medical Systems, Republic of Korea) and a portable pulsed laser system (Phocus, OPOTEK, USA). A system schematic is shown in Fig. 1b. In the portable laser system, a Q-switched Nd:YAG pump laser generates pulsed laser illumination. The wavelength of the pulsed laser is tuned by an optical parametric oscillator (OPO) in the range between 680–950 nm. The position of the OPO crystal is controlled by a PC (ThinkPad E440, Lenovo, China) with its own software. The wavelength-tuned pulsed laser is coupled to bifurcated fiber bundles that illuminate the target object. The fiber bundles are integrated with an US transducer as a handheld probe, using a custom-designed adapter. By controlling the incident laser illumination angle, the laser output from the two fiber bundles is aligned ~2 cm beneath the surface of the transducer. In the clinical US machine, acoustic waves are transmitted and received by modules controlled by the operation sequence of the system. The received acoustic waves are reconstructed to create images that are displayed in real-time. To synchronize the laser system and the US machine, a trigger signal is transmitted at every laser illumination pulse. In addition, the raw data of the acoustic waves can be extracted for post processing, such as measuring signal intensity, performing spectroscopic analysis, and reconstructing volumetric images. Figure 1c shows the control panel of the system which is the same as a typical USI system, and thus the users can control the system intuitively. Especially, a touch screen pad provides several functions such as patient management, imaging parameter setting, image and data storage, and operating program selection. Figure 1d shows operation selection interface on the touch screen pad. The users can design operation sequences of the system by writing Python scripts and easily apply their custom-designed program sequences by selecting the sequence files and touching the execution button. To acquire PA images, the operation sequence of the US machine is modified in three ways. It mutes US wave transmission, receives generated PA waves when a laser pulse illuminates the target, and applies a modified image reconstruction algorithm. The US machine provides a research platform which can implement user-designed operation sequences (Fig. 1e), which can be modified in a Python integrated development environment. All operation parameters are compiled to a sequence file (i.e., *.mat) which can be loaded by general-purpose software such as Matlab or Python. The programmable US machine loads the custom-designed sequence file and follows the operation sequence in real-time. Operation sequences for conventional USI and overlaid PAUS imaging are illustrated in Fig. 1e. In conventional USI, 12 bit analog-to-digital-converted (ADC) data are processed through beamforming, IQ demodulation, envelope detection, log compression, scan conversion, and display modules. A conventional delay and sum (DAS) beamforming algorithm is used for calculating the delay profile. For PAUSI, 12 bit ADC data are acquired at each trigger sent from the laser system, and processed as in conventional US imaging. US data and PA data are calculated in different ways, and then merged into one image. The PAI sequence is identical to the USI sequence except for the delay calculation in the beamforming algorithm. Delay profiles in PAI are calculated as a one-way trip, while in USI they are calculated as a round trip. To distinguish US and PA images, all US images are represented in grayscale, while all PA images are represented in pseudo-color. To acquire real-time images, all data are handled by parallel processing using a graphics processing unit (GPU), which can enhance processing speed dramatically. Because the transducer is composed of 128 elements and 64 channels, data from one cross sectional PA image are received twice. Consequently, the maximum frame rate of a typical PAUS image is half of the 10 Hz pulse repetition frequency of the laser system, which is much slower than the frame rate of conventional USI. Therefore, the operation sequence of PAUSI is designed to acquire US images in the interval between two PA signal-reception procedures.

### System performance

**Figure 2:**
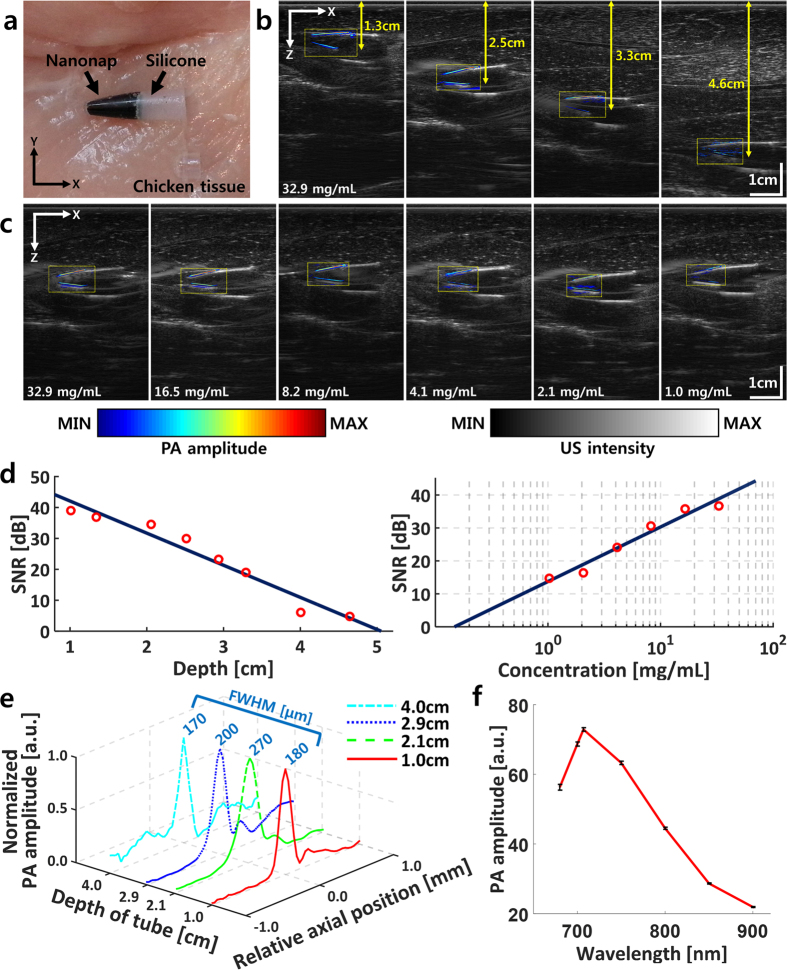
Performance of the real-time clinical PAUS imaging system. (**a**) Photograph of the nanonap sample. (**b**) Overlaid PAUS images at various depths. (**c**) Overlaid PAUS images for various concentrations. (**d**) Quantified SNR of the nanonap at various depths and concentrations. (**e**) Quantified axial resolution at various depths, measured by FWHM. (**f**) Quantified spectroscopic PA contrast of nanonap. PA, photoacoustic; US, ultrasound; SNR, signal to noise ratio; and FWHM, full width half maximum.

To test the performance of the PAUSI system in biological tissues, PA amplitudes of microtubes containing organic naphthalocyanines (nanonaps) were measured at various depths and concentrations. To eliminate attenuation of the PA waves in air, silicone sealant (RTV118, Momentive, USA) was used to fill the microtubes above the deposited nanonaps (Fig. 2a). PAUS images of nanonap-containing microtubes were acquired for various concentrations and depths in tissue. Figure 2b,c show overlaid PAUS images of the microtubes at various depths beneath chicken tissue, and in various concentrations, respectively. Raw data of both the US and PA images were acquired, and then corresponding images were reconstructed using Matlab software. All US images are represented in grayscale with the same dynamic range, whereas all the PA images are represented in pseudo-color with different dynamic ranges. The pseudo-color in each PA image is equal to the local optical absorption of the nanonap-filled microtube, while the origin of the signals is confirmed in the corresponding US image. Figure 2d shows the quantified signal-to-noise ratios (SNRs) of the nanonaps at various depths and concentrations. The SNR in decibels decreased linearly (10.38 dB/cm) with increasing depth. The maximum detectable depth was ~4.6 cm, and the noise equivalent depth was ~5.0 cm. The measured 1/e decay penetration depth was ~1.15 cm, which matched with previous reports in the literature (~1.19 cm) at an optical wavelength of 700 nm^39^. The measured noise equivalent depth was ~4.3 times the 1/e penetration depth. Therefore, a penetration depth of 3.35 cm can be achieved in human breast tissue, with a 1/e penetration depth of 0.78 cm at 683 nm^40^. Because the laser power is only one-ninth of the ANSI safety limit, the penetration depth could be increased if the laser power were increased closer to the ANSI safety limit. The SNR of the PA signals increased as the contrast agent concentration increased. The minimum clearly detectable concentration in the phantom was 1.0 mg/mL, and the noise equivalent concentration was 0.2 mg/mL. Figure 2e shows axial resolutions at various depths, measured at the boundary of each microtube. PA signals along the axial direction were normalized and fitted to a Gaussian distribution function. The axial resolution, calculated as the full width at half maximum of each Gaussian function, remained at 205 ± 45 μm despite amplitude reduction with increasing depth. The measured lateral resolutions at various depths are 1.20 ± 0.14 mm (Figure S1). In addition, the spectroscopic PA contrast of the nanonap is shown in Fig. 2f. The nanonaps generated the greatest PA amplitude at a wavelength of 707 nm, which matched the optical absorption characteristics of the material (Figure S2)^6^.

### Testing various ultrasound transducers

**Figure 3:**
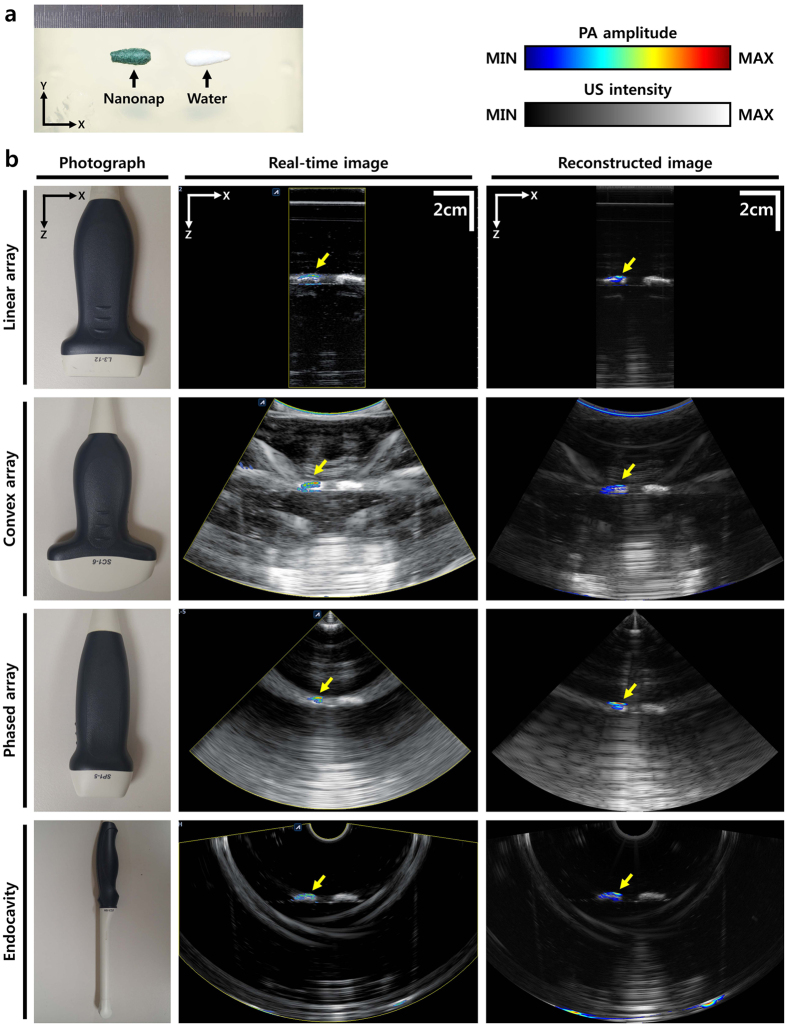
PAUS images of a phantom, using various transducers. (**a**) Photograph of the phantom. (**b**) Photographs, real-time PAUS images, and reconstructed PAUS images acquired with linear array, convex array, phased array, and endocavity transducers. PA, photoacoustic; and US, ultrasound.

A gelatin-lipid phantom was prepared to confirm the imaging capability of the system with various transducers. Two samples, a nanonap contrast agent and a water control, were placed 4 cm under the surface of the phantom (Fig. 3a). Like a conventional US imaging machine, the programmable clinical US machine can utilize various US transducers. We acquired PAUS images of a phantom using a linear array (L3-12, Alpinion Medical Systems, Republic of Korea), a convex array (SC1-6, Alpinion Medical Systems, Republic of Korea), a phased array (SP1-4, Alpinion Medical Systems, Republic of Korea), and an endocavity transducer (EC3-10H, Alpinion Medical Systems, Republic of Korea). Photographs, real-time PAUS images, and reconstructed PAUS images from each transducer are shown in Fig. 3b. All US images are represented in grayscale, and all PA images are represented in pseudo-color. Both samples are clearly visible in the US images because they have identical acoustic properties. In contrast, only the nanonap sample is visible in the PA images due to its high optical absorption in the near infrared. These results show proof-of-principle that overlaid PAUS images can provide both structural information as well as functional information. In addition, the PAUSI capacity is fully compatible with several different transducers. Therefore, by selecting a suitable transducer, the PAUSI system can readily be applied to a variety of clinical applications where USI is already used, including thyroidal, hepatic, cardiac, intestinal, and ovarian diseases.

### *In vitro* vascular mimicking phantom imaging

**Figure 4:**
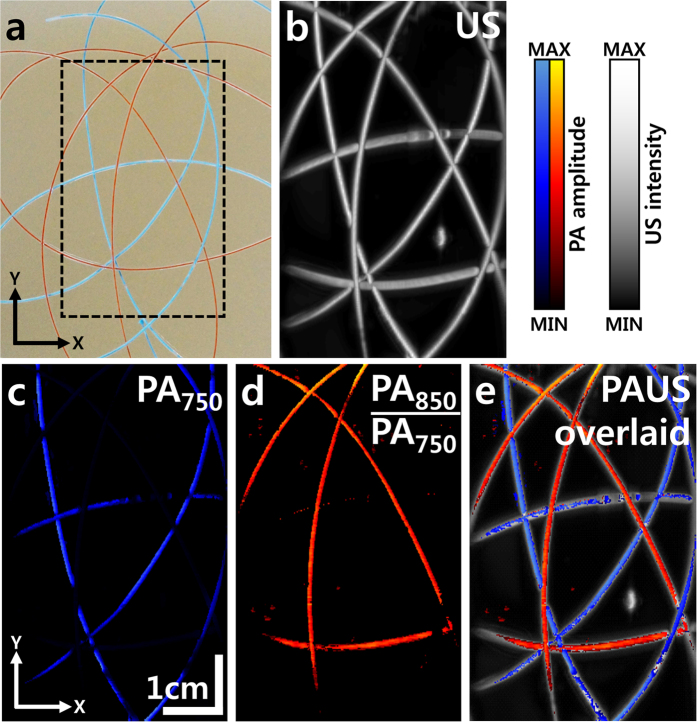
PAUS images of a vasculature mimicking phantom. (**a**) Photograph of the phantom. The dashed rectangle marks the imaging region. (**b**) US MAP image of the phantom. (**c**) PA MAP image of the phantom at an excitation wavelength of 750 nm. (**d**) Proportional PA MAP image (PA_850_/PA_750_) of the phantom. (**e**) Overlaid PAUS MAP image of the phantom. PA, photoacoustic; US, ultrasound; PA_750_, PA signal at 750 nm excitation wavelength; PA_850_, PA signal at 850 nm excitation wavelength; and MAP, maximum amplitude projection.

To verify the volumetric imaging capability of the developed system, we imaged a vasculature mimicking phantom. (Fig. 4a). Because a planar image is acquired at one position, scanning the handheld probe in the elevational direction is required to acquire a volumetric image. The volumetric images are displayed in two-dimensional planes as maximum amplitude projections (MAP). US, PA, and overlaid PAUS MAP images of the phantom are shown in Fig. 4b–e. In the PA MAP images, the optical absorption characteristics of each tube are distinguishable between the two excitation wavelengths, but not in the US MAP images. The PA signals of the blue tubes are dominant at 750 nm, while the PA signals of both tubes are similar at 850 nm (Figure S3). To distinguish the red tubes from the blue tubes, the proportional difference (PA_Red_ = PA_850_/PA_750_) between the two excitation wavelengths was calculated for each pixel of the image. In addition, PA and US MAP images are overlaid in one image. The US image is represented in grayscale, whereas PA images are represented in corresponding blue and red. The resulting images both visualize the optical absorption characteristics of the vasculature samples in the phantom and provide structural information.

### *In vivo* animal imaging

**Figure 5:**
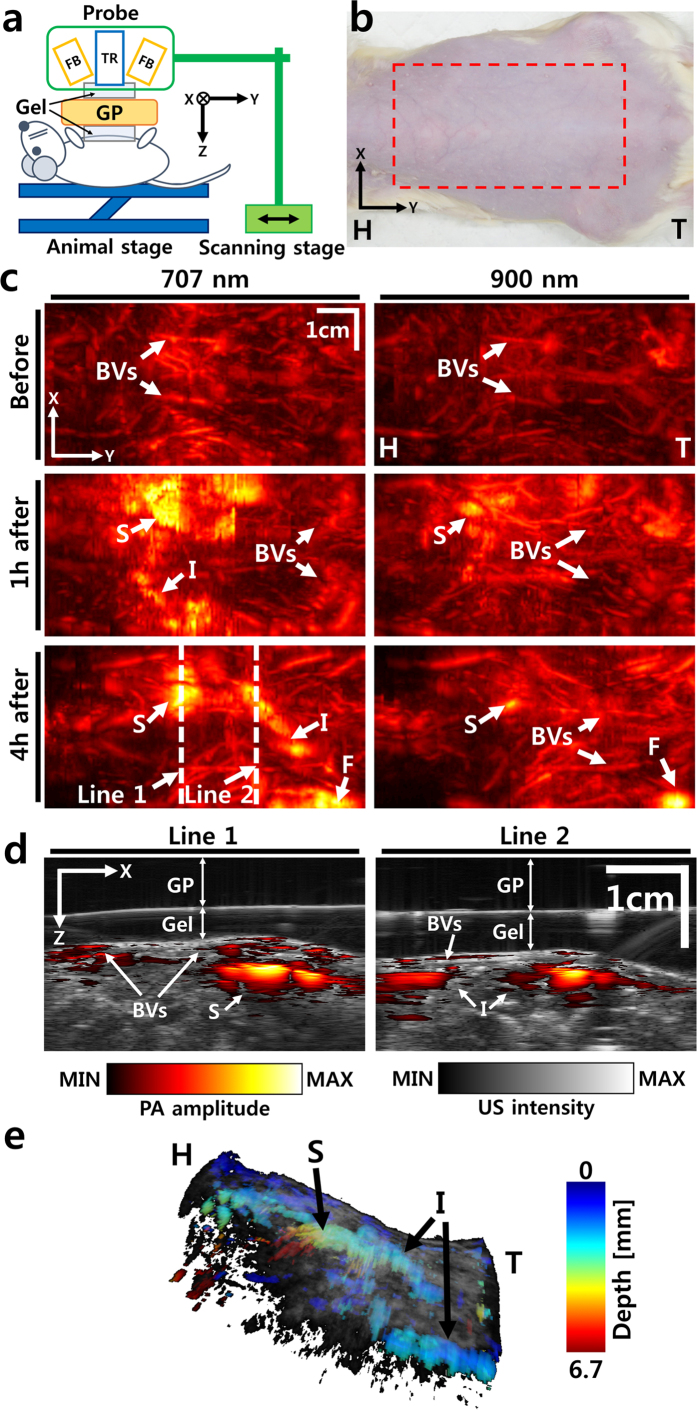
Noninvasive *in vivo* PAUS imaging of a rat GI tract. (**a**) Schematic of experimental setup. (**b**) Photograph of the rat and imaging region (the red dashed rectangle). (**c**) *In vivo* spectroscopic (707 and 900 nm) PA MAP images of rat GI tract before, 1 hour after, and 4 hours after oral administration of nanonaps. (**d**) Cross sectional overlaid PAUS images at two white dashed lines in c. (**e**) Depth resolved volumetric PAUS image of the rat 4 hours after oral administration of the nanonap. PA, photoacoustic; US, ultrasound; GI, gastro intestinal; MAP, maximum amplitude projection; H, head; T, tail; FB, fiber bundle; TR, ultrasound transducer; GP, gelatin pad; BVs, blood vessels; S, stomach; I, intestine; and F, feces.

To verify the imaging capability of the system in living tissue, volumetric PAUS images of the GI tract in a rat were acquired *in vivo*. Figure 5a,b show a schematic and photograph of the rat and the imaging area (red dashed rectangle). Figure 5c shows PA MAP images of the GI tract in the rat. Before administration of nanonaps, blood vessels in the rat are clearly visible. Interestingly, the descending aorta is visualized as well as small blood vessels near the skin surface. After administration of nanonaps, the contrast position and flow is clearly visible through the stomach and intestine of the rat. The spectroscopic characteristics of the GI tract are well matched with the optical absorption characteristics of the nanonaps. In PA images at a wavelength of 707 nm, the contrast of the nanonaps is dominant, and blood vessels are concealed. On the other hand, in PA images at 900 nm, blood vessels are revealed because the contrast for this particular type of nanonap is significantly decreased at that wavelength (Figure S2). Figure 5d shows cross sectional PAUS images at the two white dashed lines in Fig. 5c. The positions of the stomach and intestines, as well as small surface blood vessels, can be observed. Figure 5e is a volumetric PAUS image at 4 hours after oral nanonap administration. The volumetric image is visualized by 3-dimensional rendering software (Amira, Visualization Science Group, France). A movie from the volumetric image is available online (Movie S1). In the volumetric image, the US image is represented in grayscale, and the depth resolved PA image is represented in pseudo-color. After all experiments, the rat was sacrificed and dissected to confirm the position of the internal organs in the PAUS images (Figure S4). The position and optical characteristics of the internal organs matched well with the PAUS images. The GI tract appeared green due to the color of the residual nanonaps.

### *In vivo* human imaging

**Figure 6:**
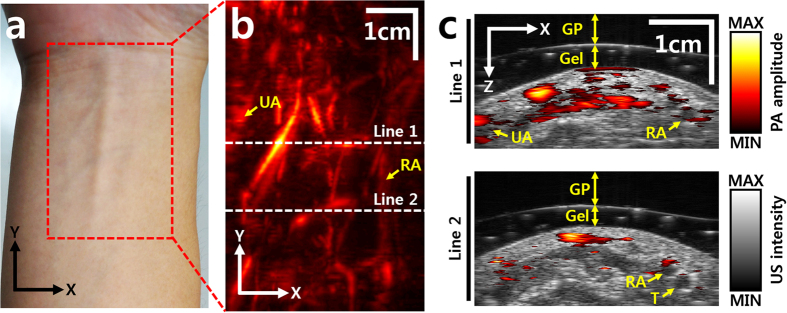
*In vivo* PA images of a human forearm. Photograph (**a**) and PA MAP (**b**) image of a human right forearm. The red dashed rectangle outlines the imaging region. (**c**) Cross sectional overlaid PAUS images at the two white dashed lines in b. GP, gelatin pad; UA, ulnar artery; RA, radial artery; T, tendon; PA, photoacoustic; US, ultrasound; and MAP, maximum amplitude projection.

To confirm the system capability for human imaging, volumetric PAUS images of a human right forearm were acquired *in vivo* (Fig. 6a). The red dashed rectangle in Fig. 6a shows the imaging area. To enhance acoustic impedance matching, a 5 mm thick gelatin pad and US gel (Ecosonic, SANIPIA, Republic of Korea) were applied between the transducer and skin. Figure 6b is a volumetric PA MAP image, in which blood vessels in the forearm are clearly visible. Figure 6c shows depth resolved cross sectional PAUS images at the two white dashed lines in Fig. 6b. The ulnar and radial arteries are visible in the cross sectional images^41^. The cross -sectional PAUS images of the human forearm obtained with various transducers are shown in Figure S5. From the figures, it is apparent that the developed clinical PAUSI system can provide high-quality imaging of human tissues *in vivo* and is ready for further use in clinical research applications.

## Discussion

A clinical real-time PAUSI system has been implemented by integrating an FDA-approved programmable clinical USI machine and a portable laser system. The operation sequences of the US machine were modified to acquire both PA images and US images in real-time. Then, PA, US, and overlaid PAUS images are displayed in real-time. Because various US transducers can be applied to acquire PAUS images, users can select the most appropriate transducer for their specific purpose. The volumetric imaging capability of the system was verified by acquiring PAUS images of a vasculature mimicking phantom, a contrast-enhanced rat GI tract *in vivo*, and a human forearm *in vivo*. The developed system is the first PAUSI system to be based on a conventional clinical US machine, and provides conveniences such as handheld operation, an intuitive user interface, complete portability, and real-time imaging. The developed system is completely programmable on the basis of the FDA-approved clinical US imaging system and the laser system provides a wide tuning range in the NIR region. Therefore, the users can modify and apply this system to their specific clinical research interests. The developed PAUSI system is well-suited for both clinical practice as well as preclinical research.

## Methods

### Organic nanoformulated naphthalocyanines (nanonaps)

To test the performance of the PAUSI system in live animals, nanonaps were used as contrast agents. Nanonaps were prepared as previously reported^6^, using chemicals obtained from Sigma-Aldrich. Briefly, 40 mg of ZnBNc (Zinc- 2,11,20,29-tetra-tert-butyl-2,3-naphthalocyanine) was dissolved in 200 mL of methylene chloride, followed by slow addition of the solution to 1 L of 10% (w/v) Pluronic F127 solution. After being stirred overnight and spun at 3,500× g for 10 min, the supernatant was subjected to a surfactant-stripping process using membrane filtration (1501008VS, Sartorius Vivaflow, Germany) via a peristaltic pump (Masterflex L/S, Cole-Parmer, USA) and tubing (Masterflex 6434-16, Cole-Parmer, USA) at a temperature close to 0 °C (achieved by an ice bath). Purified nanonaps were concentrated by centrifugal filtration (UCF9-100-24, Fisher Scientific, USA). The obtained nanonaps dispersion had a maximum absorption at 707 nm, with a size of 20 nm, as determined by dynamic light scattering (Nanobrook 90Plus PALS, Brookhaven, USA).

### Performance test

The nanonap-containing microtubes were placed in chicken breast tissues to mimic optical diffusion in living biological tissues. The experiments were followed as previously reported^11^, a thick (~8 cm) layer of chicken breast tissue was placed under the microtubes to prevent PA signal reflection, and then more chicken breast tissue layers were placed over the microtubes to measure the amplitude of PA signals at various depths (1.0, 1.3, 2.1, 2.5, 2.9, 3.3, 4.0, and 4.6 cm). The depths of the top and bottom surfaces of the microtubes were measured in the US images. In addition, the amplitudes of the PA signals from the microtubes containing various concentrations (1.0, 2.1, 4.1, 8.2, 16.5, and 32.9 mg/mL) of nanonaps were acquired at a tissue depth of ~2 cm. We photoacoustically imaged a human hair with a diameter of 93 μm to measure the lateral resolution. To generate PA waves, a laser excitation wavelength of 707 nm was used, which is the peak absorption wavelength of the type of nanonap used in this study. We acquired 10 PA signals at each position and concentration, and then averaged the signals to increase SNRs. The output energy of the laser was 10.1 mJ/cm^2^, which is only one-ninth of American National Standards Institute (ANSI) safety limit (20 mJ/cm^2^)^42^.

### Testing various ultrasound transducers

To enhance the optical scattering properties of the phantom, 5% intralipid was added to the phantom. A wavelength of 707 nm with an output power of 10.1 mJ/cm^2^ was used for each image. Real-time PAUS images were acquired by four different types of US transducers (e.g., linear (3–12 MHz), convex (1–6 MHz), phased (1–5 MHz), and endocavity (3–10 MHz) arrays).

### *In vitro* vascular mimicking phantom imaging

A loose mesh of silicone tubes (508-001, Dow Corning, USA), filled with two types of optical absorbers (blue and red paint), was positioned inside the gelatin phantom. The optical absorption coefficients of these two paints are measured in Figure S3. The inner and outer diameters of the tubes were 0.30 and 0.64 mm, respectively. To enhance optical scattering, 5% intralipid (i.e., an optical absorption coefficient of 0.01 cm^−1^ and optical scattering coefficient of 400 cm^−1^)^43^ was added to the phantom. Two laser excitation wavelengths, 750 and 850 nm, were used to enable spectroscopic analysis. To acquire volumetric images, a motorized linear scanner (STM-1-USB, ST1, Republic of Korea) moved the handheld probe along the elevational (y) direction. The scanning range, step size, and scanning speed were 60 mm, 0.2 mm, and 2 mm/s, respectively.

### *In vivo* animal imaging

All animal experimental procedures were performed in accordance with protocols approved by the institutional animal care and use committee of Pohang University of Science and Technology (POSTECH). To acquire volumetric images of gastro-intestinal (GI) tracts, we used healthy ten-week-old female Sprague-Dawley rats weighing 200–250 g. Each rat was initially anesthetized using a mixture of ketamine (85 mg/kg) and xylazine (15 mg/kg), and then its abdominal hair was removed with depilatory lotion. During the experiments, anesthesia was maintained by a vaporized isoflurane (1 L/min of oxygen and 0.75% isoflurane) gas system (VIP3000, Midmark, USA). The rat was placed on a custom designed small animal imaging setup to acquire volumetric PAUS images (Fig. 5a). The handheld probe was fixed above the rat and moved in the elevational (y) direction by a motorized linear scanner with a scanning range of 75 mm, a step size of 0.5 mm, and a scanning speed of 2.5 mm/s. A gelatin pad and US gel were applied between the probe and the rat to enhance acoustic impedance matching. To enhance the optical absorption contrast of the GI tract in the rat, 400 μL of organic nanonaps with a concentration of 15 mg/mL were orally injected. Volumetric PAUS images were acquired before, 1 hour after, and 4 hours after oral administration of the nanonaps. Laser excitation wavelengths of 707 and 900 nm were used to acquire the volumetric PAUS images. The output energy of the laser at 707 and 900 nm were 10.1 and 10.5 mJ/cm^2^, respectively, much less than corresponding ANSI safety limits of 20 and 40 mJ/cm^2^ 42. The rat was awakened between the imaging sessions to make the nanonaps were flowed by enterokinesia. After all imaging experiments, the rat was sacrificed with an overdose of pentobarbital, then dissected and photographed to validate the *in vivo* imaging results.

### *In vivo* human imaging

All human experimental procedures were conducted in accordance with protocols approved by the institutional review board of POSTECH. To acquire volumetric images of a human forearm, we recruited healthy volunteers. Informed consents were received from all the volunteers under sufficient explanation about the experiments. The volunteers fixed their forearm on an imaging stage while an imaging probe scanned the imaging region. To enhance acoustic impedance matching, conventional US gel was applied between the forearm and the probe. A volumetric PAUS image of the forearm was acquired by elevational scanning (y direction) using a motorized linear scanner with a range of 60 mm, a step size of 0.4 mm, and a scanning speed of 2 mm/s. An excitation wavelength of 850 nm was used to acquire the volumetric PAUS images. To prevent eye damage by accidental laser illumination, all the volunteers and experimenters wore safety glasses and flame resistant gowns. The output energy of the pulse laser was 10.7 mJ/cm^2^ at 850 nm, much less than the ANSI safety limit of 40 mJ/cm^2 ^42.

## Additional Information

**How to cite this article**: Kim, J. *et al*. Programmable Real-time Clinical Photoacoustic and Ultrasound Imaging System. *Sci. Rep*. **6**, 35137; doi: 10.1038/srep35137 (2016).

## Supplementary Material

Supplementary Information

Supplementary Movie S1
