# Supplementary material for: Programmable Real-time Clinical Photoacoustic and Ultrasound Imaging System
Source: Sci Rep. 2016 Oct 12;6:35137. doi: 10.1038/srep35137 (PMC5059665; doi:10.1038/srep35137)
Supplement: Supplementary Information [file srep35137-s1.pdf]

Supplementary Information

Programmable Real-time Clinical Photoacoustic and Ultrasound Imaging System

Jeesu Kim[1], Sara Park[1], Yuhan Jung[1], Sunyeob Chang[2], Jinyong Park[2], Yumiao Zhang[3], Jonathan F. Lovell[3], and Chulhong Kim[1,*].

[1]Departments of Creative IT Engineering and Electrical Engineering, Pohang University of Science and Technology (POSTECH), 77 Cheongam-Ro, Nam-Gu, Pohang, Gyeongbuk, 37673, Republic of Korea.

[2]Alpinion Medical Systems, 72 Digital-Ro 26-Gil, Guro-Gu, Seoul, 08393, Republic of Korea.

[3]Department of Biomedical Engineering, University at Buffalo, The State University of New York, Buffalo, NY 14260, United States of America

[*]Corresponding author: chulhong@postech.edu

E-mail address:

Jeesu Kim: hybridjs@postech.ac.kr

Sara Park: sarapark@postech.ac.kr

Yuhan Jung: atcham@postech.ac.kr

Sunyeob Chang: sunyeob.chang@alpinion.com

Jinyong Park: jinyong.park@alpinion.com

Yumiao Zhang: yumiaozh@buffalo.edu

Jonathan F Lovell: jflovell@buffalo.edu

Chulhong Kim: chulhong@postech.edu

Address correspondence to: Chulhong Kim, PhD, Bio Optics and Acoustics Laboratory, Pohang University of Science and Technology, 77 Cheongam-Ro, Nam-Gu, Pohang, Gyeongbuk, 37673, Republic of Korea. Phone: +82-54-279-8805. Fax: +82-54-279-8899. E-mail: chulhong@postech.edu

Table S1. Specifications of commercial photoacoustic/ultrasound imaging systems. PRF, pulse repetition frequency; PA, photoacoustic; and FDA, United States Food and Drug Administration.

System	Laser source		Imaging system						
	PRF [Hz]	Wavelength [nm]	PA frame rate [Hz]	PA penetration depth [cm]	Ultrasound frequency for PA imaging [MHz]	Transducer for PA imaging	Programmable	User interface for PA imaging	FDA approved
**Verasonics**	Laser dependent	Laser dependent	Laser dependent	Laser dependent	Transducer dependent	Linear	Yes	No	No
**VevoLAZR (Visualsonics)**	20	680-970	5	1.5	21	Linear	No	Yes	No
**Nexus 128 (Endra)**	20	680-950	Reconstruction time: < 1 minutes	1.5	5	Helical	No	Yes	No
**MSOT Acuity (iThera)**	10 or 100	680-980	Up to 50	1-3	2.5, 10	2D array	No	Yes	No
**Louisa3D (TomoWave)**	10	532, 730, 850, 1064	–	–	4	Arc	No	Yes	No
**PA mammography (Canon)**	10	680-950	Total scan time: 12 seconds	–	2	Hemisphere	No	Yes	No
**Twente PA mammoscope (Twente)**	10	1064	–	–	1	Flat	No	Yes	No
**Imagio (Seno medical)**	–	–	–	–	–	Linear	No	Yes	No
**Prodigy (S-Sharp)**	–	–	–	–	–	–	Yes	No	No
**Sonix Touch Q+ (BK)**	–	–	10	3	7	Linear	Yes	No	Ultrasound only
**Z.One PRO (Zonare)**	–	–	10	1.5	11.25	Linear	Yes	No	Ultrasound only
**POSTECH**	10	680 – 950	5	4.5	7.5<br>3.5<br>2.5<br>6.5	Linear,<br>Convex,<br>Phased,<br>Endocavity	Yes	Yes	Ultrasound only

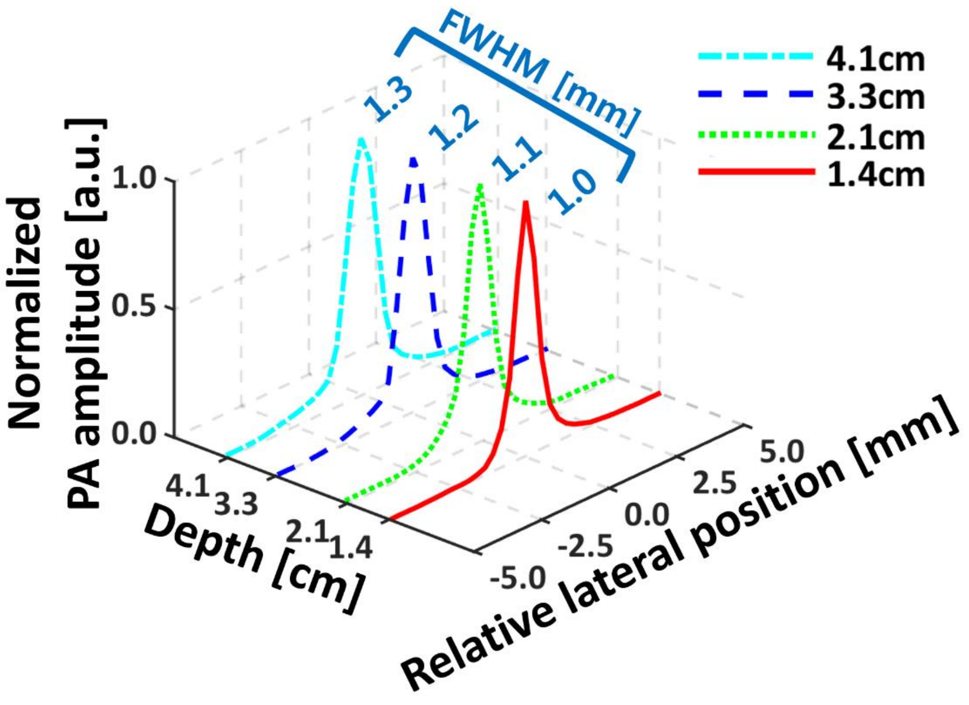

Figure S1. Quantified lateral resolution at various depths, measured by FWHM. PA, photoacoustic; and FWHM, full width at half maximum.

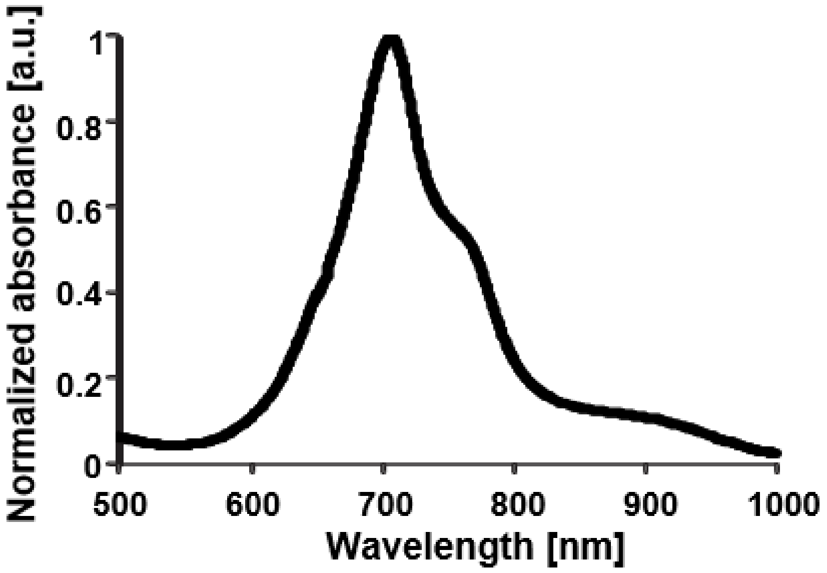

Figure S2. Normalized optical absorbance spectrum of the nanonap. The peak absorption wavelength is 707 nm.

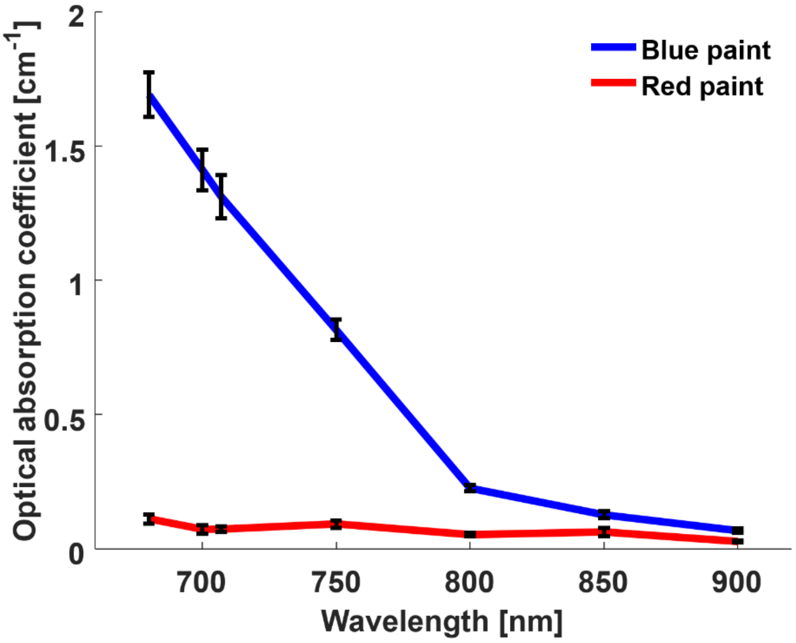

Figure S3. Optical absorption coefficients of blue and red paints.

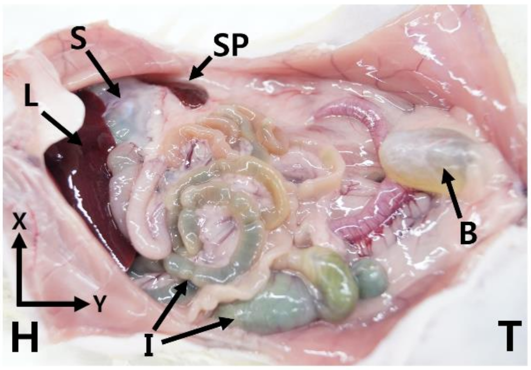

Figure S4. Photograph of the dissected rat acquired after *in vivo* imaging. The GI tract is dyed in green, which is the color of the nanonap. GI, gastrointestinal, H, head; T, tail; L, liver; S, stomach; I, intestine; and B, bladder.

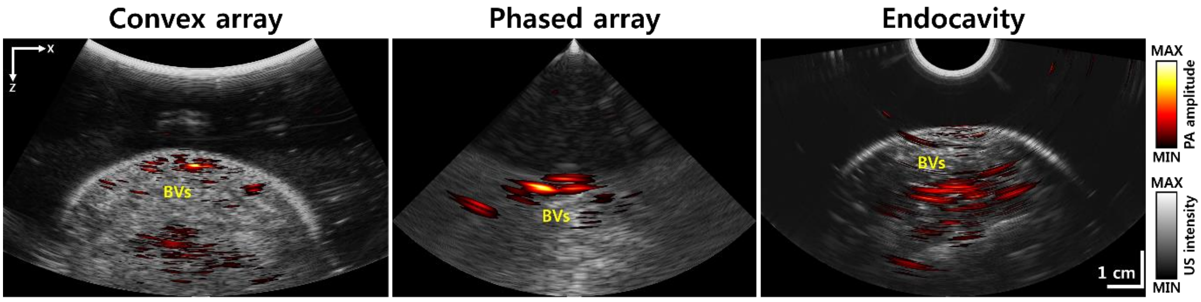

Figure S5. *In vivo* overlaid photoacoustic/ultrasound images of a human forearm acquired with convex array, phased array, and endocavity transducers. BV, blood vessel.

Movie S1. Movie form the volumetric images of the gastro-intestinal tract in a rat *in vivo*.
